# Does Locoregional Chemotherapy Still Matter in the Treatment of Advanced Pelvic Melanoma?

**DOI:** 10.3390/ijms18112382

**Published:** 2017-11-09

**Authors:** Stefano Guadagni, Giammaria Fiorentini, Marco Clementi, Giancarlo Palumbo, Paola Palumbo, Alessandro Chiominto, Stefano Baldoni, Francesco Masedu, Marco Valenti, Ambra Di Tommaso, Bianca Fabi, Camillo Aliberti, Donatella Sarti, Veronica Guadagni, Cristina Pellegrini

**Affiliations:** 1 Department of Applied Clinical Sciences and Biotechnology, University of L’Aquila, 67100 L’Aquila, Italy; francesco.masedu@cc.univaq.it (F.M.); marco.valenti@cc.univaq.it (M.V.); cristina.pellegrini@cc.univaq.it (C.P.); 2 Department of Oncology and Hematology, Ospedali Riuniti Marche Nord, 61121 Pesaro, Italy; g.fiorentini@alice.it (G.F.); d.sarti@fastwebnet.it (D.S.); 3 Department of Life, Health and Environmental Sciences, University of L’Aquila, 67100 L’Aquila, Italy; marco.clementi@univaq.it (M.C.); giancarlo.palumbo@cc.univaq.it (G.P.); paola.palumbo@univaq.it (P.P.); alessandro.chiominto@univaq.it (A.C.); stefano.baldoni@univaq.it (S.B.); ambra.ditommaso@graduate.univaq.it (A.D.T.); biancafabi@gmail.com (B.F.); 4 Department of Radiology, Institute for the Research and Treatment of Cancer, 35128 Padova, Italy; camillo.aliberti@ioveneto.it; 5 Department of Physiology and Pharmacology, Cumming School of Medicine, University of Calgary, Calgary, AB T2N 4N1, Canada; vguadagn@ucalgary.ca

**Keywords:** melanoma, *BRAF*, Melphalan, pelvic perfusion, hypoxia, stopflow

## Abstract

Pelvic Melanoma relapse occurs in 15% of patients with loco regional metastases, and 25% of cases do not respond to new target-therapy and/or immunotherapy. Melphalan hypoxic pelvic perfusion may, therefore, be an option for these non-responsive patients. Overall median survival time (MST), stratified for variables, including BRAF V600E mutation and eligibility for treatments with new immunotherapy drugs, was retrospectively assessed in 41 patients with pelvic melanoma loco regional metastases. They had received a total of 175 treatments with Melphalan hypoxic perfusion and cytoreductive excision. Among the 41 patients, 22 (53.7%) patients exhibited a wild-type *BRAF* genotype, 11 of which were not eligible for immunotherapy. The first treatment resulted in a 97.5% response-rate in the full cohort and a 100% response-rate in the 22 wild-type *BRAF* patients. MST was 18 months in the full sample, 20 months for the 22 wild-type *BRAF* patients and 21 months for the 11 wild-type *BRAF* patients not eligible for immunotherapy. Melphalan hypoxic perfusion is a potentially effective treatment for patients with pelvic melanoma loco regional metastases that requires confirmation in a larger multicenter study.

## 1. Introduction

Cutaneous melanoma is one of the most aggressive treatment-resistant cancers [1]. Proto-oncogenes and tumor suppressor genes involved in the Mitogen-Activated Protein Kinase (MAPK) pathway have been implicated in the molecular pathogenesis of cutaneous melanoma, with activating mutations in *BRAF* (v-Raf murine sarcoma viral oncogene homolog B) and *NRAS* (neuroblastoma RAS viral oncogene homolog) encountered in approximately 70% of all melanomas [1]. Somatic *BRAF* mutation in codon 600 of exon 15 occurs in 40–50% of cutaneous melanomas, with V600E the most common mutation. BRAF^V600E^ is now recognized as a validated therapeutic target, although acquired resistance is almost universal [2].

Recently, novel immunotherapies that target negative immune checkpoint molecules have gained a major interest in the treatment of melanoma [2]. Therefore, in the last six years, options for treatment of advanced melanoma patients have significantly changed, thanks to new target therapy and immunotherapy. The new era of effective systemic therapy, also involves patients with pelvic locoregional metastases without lesions in the legs, which are approximately less than 2% of all malignant melanoma patients [3,4].

Unfortunately, target therapy provides a significant overall median survival improvement in only 50% of patients who carry the BRAF^V600E^ mutation, with salvage immunotherapy, following discontinuation of targeted therapy, frequently unsatisfactory [5,6]. Furthermore, these new immunotherapies are effective in only 45% of wild-type *BRAF* melanoma patients, associated with overall median survival times ranging from 11 to 20 months, with adverse events observed in 4–25% of patients [7,8,9].

An effective treatment for melanoma patients with loco-regional pelvic metastases, who do not respond to target therapy and/or new immunotherapy, remains an important area for clinical research. A recent review has examined the role of surgery and loco-regional chemotherapy in the management of in-transit disease, in the era of effective systemic therapy [10]. A decade ago, standard treatment for patients with loco-regional melanoma metastases resulted in a median survival time of approximately eight months [11,12] and high complex regional chemotherapy procedures containing Melphalan were considered to have potential to improve clinical outcome [4,13,14,15,16]. However, techniques were not standardised and results varied according to the experience of each institution. Since then, more feasible procedures associated with lower morbidity and fewer adverse effects have been developed, with particular emphasis on the use of interventional radiology [14]. An important question however, remains to be answered, and that is whether loco-regional chemotherapy, performed by the surgical or percutaneous approach, still has a place in the treatment of advanced stage melanoma?

In this report, we present a retrospective study of the efficacy of Melphalan hypoxic perfusion in patients with pelvic metastatic melanoma stratified for prognostic factors, including *BRAF*.

## 2. Results

### 2.1. Patients Characteristics

A total of 41 melanoma patients with metastatic lesions were included in this study (13 males and 28 females). Mean patient age (±SD, standard deviation) was 63.9 years (±13.6), mean male age was 58.2 years (±14.7) and mean female age was 66.3 years (±12.5). Seven lymph node negative patients with loco-regional metastases were classified as stage IIIB and 24 lymph node positive patients were classified as stage IIIC. The ten stage IV patients were classified by the presence of concurrent metastases to the lungs (four patients), bones (four patients) or abdomen (two patients).

### 2.2. Treatments

A total of 175 perfusions were performed, including 52 surgical procedures and 123 percutaneous procedures. The mean (±SD) number of treatments received by each patient was 4.3 (±3.1) and the median number of treatments received was four (range 2–5). Contemporary palliative cytoreduction was performed in 35 patients (85.4%). With respect to hospitalization, the median length of post-surgical perfusion recovery was 8.8 days, which was significantly longer (*p* < 0.01) than following percutaneous perfusion (4.7 days).

Patients did not experience any technical (i.e., balloon rupture), hemodynamic, or vascular complications, and no deaths occurred during the 175 procedures or during the post-operative period. Hematological toxicity, resulting in the termination of treatment, occurred in three patients following the 4th procedure (9.7%) and in a single patient following the 14th procedure. Procedure-related complications and toxicities are listed in Table 1.

### 2.3. BRAF Mutational Status

*BRAF* gene mutational analysis in the 41 metastatic melanoma tissues, identified the V600E BRAF mutation in 19 metastases (19/41, 46.3%), with 22 samples (22/41, 53.7%) characterized as wild-type *BRAF*. Eleven (11/41, 26.8%) wild-type *BRAF* patients were not suitable for immune check-point therapy, three of which (3/41, 7.3%) were in disease progression after Ipilimumab immunotherapy and 8 of which (19.5%) were ineligible due to hepatitis C (4 patients; 9.7%), human immunodeficiency virus-HIV (one patient; 2.4%) and active inflammatory bowel disease (three patients; 7.3%).

### 2.4. Tumor Responses

Tumor response was related primarily to perfusion, with minimal contribution made by additional surgical excision. In the full sample cohort, the overall response rate after the first treatment was 97.5%, with a complete response observed in four patients (9.7%), a partial response observed in 36 patients (87.8%) and stable disease observed in one patient (2.4%). No evidence of disease progression was detected within 30 days, following initial treatment. In patients who underwent more than three treatments the overall response rate was 27.3%.

In the 22 wild-type *BRAF* patients, the overall response rate following the initial treatment was 100%, with two complete responses (9.1%) and 20 partial responses (90.9%), recorded. In wild-type *BRAF* patients who underwent more than three treatments, the overall response rate was 33.3%.

With respect to the 11 wild-type *BRAF* patients that were not eligible or non-responsive to immunotherapy, two patients exhibited a complete response (18.2%) and nine patients exhibited a partial response (81.8%), following the initial treatment. Partial responses were recorded for 10% of patients following the second treatment, 11.1% of patients following the 3rd treatment and 16.7% of patients following the fourth treatment.

### 2.5. Survival

**Figure 1:**
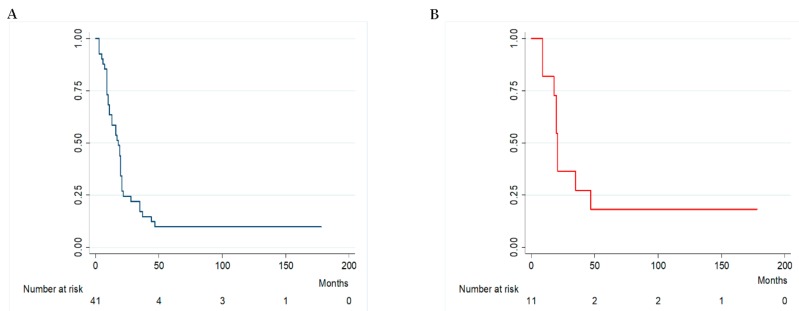
Kaplan-Meyer survival. (**A**) overall; (**B**) 11 wild-type *BRAF* patients not eligible for check-point therapy or non-responsive for progression or adverse events.

The overall MST for this patient cohort was 18 months (range 9–22) (Figure 1A), with a mean survival time of 27.6 (±35.7) months. The one-year, three-year and five-year survival rates were 63.4%, 17.1% and 9.7%, respectively and the overall median progression free survival (PFS) was 15.5 months (range 6–21), with a mean of 25.7 (±36.3) months.

Table 2 shows factors associated with survival, including gender, age, stage of disease, number and dimension of nodules, melanin cellular pigmentation, mitosis, associated excision, number of treatments and *BRAF* status.

**Figure 2:**
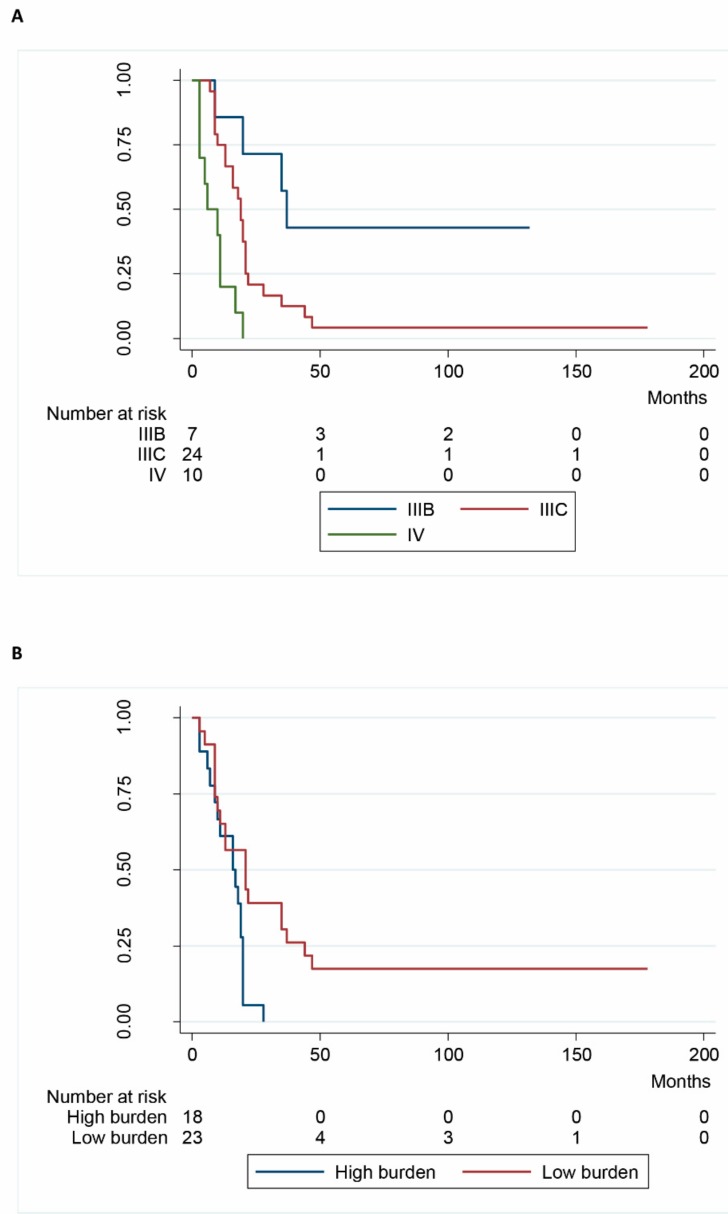
Kaplan-Meyer survival. (**A**) Stratified by Stage; (**B**) stratified by Burden.

Disease stage (*p* = 0.001) (Figure 2A), and “burden” (*p* = 0.005) (Figure 2B) significantly affected survival, whereas gender, age, mitosis, melanin pigmentation, associate excision, number of treatments, and *BRAF* V600E status did not.

In 22 wild-type *BRAF* patients, the MST was 20 months (range 11–21), with a mean survival time of 31.3 (±41.9) months. Gender, the number of treatments and nodule number and dimension (burden) all had an impact on survival that could not be evaluated statistically due to the small number of patients in these sub-groups. The median PFS was 17.7 months (range 9.5–20), with a mean of 29.4 (±42.5) months.

In the 11 wild-type *BRAF* patients that were not eligible or non-responsive to checkpoint therapy, the MST (Figure 1B) was 21 months (range 18–47), with a mean of 46.4 (±55.7) months. The median PFS for this group was 20 months (range 16.5–45), with a mean of 44.4 (±56.8) months.

### 2.6. Follow-Up

Median follow-up was 18 months (range 9–22), with a mean of 27.6 (±35.7) months. Among the 41 patients studied, four (9.8%) are still alive without evidence of disease after 76, 109, 132 and 178 months, whereas the remaining 37 (90.2%) have deceased as a consequence of melanoma. Therapy was interrupted in one patient due to Melphalan allergy, following the 10th treatment and another patient developed a brain metastasis six-years after the last perfusion. This patient was referred for surgical excision and remains disease-free after nine years.

With respect to the 20 patients who interrupted treatment due to disease progression, the median MST was 10 months, with a mean of 12.1 (±8.6) months, which was significantly shorter (*p* < 0.01) than the MST of the 10 patients who interrupted treatment due to worsening condition (median 20; mean 27.5 ± 11.6 months) or the seven patients who retired the consent (median 20; mean 17.0 ± 5.6 months; *p* < 0.04).

Disease progression in the pelvis was detected in 17 stage IIIC patients (one of which also developed distant site relapse) and in two stage IIIB patients. Distant site relapse was observed in two stage IIIB patients, seven stage IIIC patients and in 10 stage IV patients (one also with pelvic recurrence).

## 3. Discussion

In this retrospective study, we present evidence that demonstrates the potential efficacy of Melphalan hypoxic pelvic perfusion in patients with pelvic and/or inguinal loco regional melanoma metastases.

Prior to 2013, pelvic perfusion was an option for patients with loco regional melanoma metastases who were non-responsive to standard treatments. Within the last four years, immunotherapy with checkpoint inhibition and MAPK pathway targeted inhibitory therapy have led to important improvements in patient outcomes and has become the first line of therapy. Reports suggest, however, that up to 25% of melanoma patients may not respond to new target and immunotherapeutic drugs [17]. In our retrospective study, melanoma patients received repeated Melphalan hypoxic pelvic perfusions associated with cytoreductive excision between 2002 and 2013. This cohort had several interesting characteristics: (1) patients with loco regional pelvic melanoma metastases with or without leg lesions below mid-thigh level, represent a very rare category (enrollment of three/four patients per year) and the small sample size was a deliberate choice from a single institute in order to minimize procedural and technical bias, consistent with similar sample sizes in previous reports [13,14,15,16]; (2) in all 41 patients, lymphocyte invasion into metastatic melanoma tissues was not detected. This condition has been reported recently to associate with melanoma resistance to anti-PD1 antibody therapy [17]. Furthermore, melanomas did not exhibit desmoplasia with malignant spindle cells (DM). The prognosis for melanoma with DM is controversial with a recent report indicating a similar survival rate for case-matched patients with or without DM [18].

Molecular analysis of metastatic melanomas identified a BRAF^V600E^ mutation-rate of 46.3%. This frequency is similar to that commonly found for primary tumours in large series of cases [19] and in other Italian cohorts [20]. It remains unclear, however, whether the primary tumour *BRAF* mutation status is retained in metastases and we are unable to add anything more to this debate, in this study.

Among the 22 wild-type *BRAF* patients, 11 were not eligible for new immunotherapeutic drugs or had therapy interrupted due to disease progression or adverse events. A recent review discusses the reasons for which many patients seen in routine clinical practice do not qualify for immune checkpoint inhibitor clinical trials and have been seriously underrepresented in new immunotherapy trials [21].

In this study, Melphalan mono-therapy was chosen for this patient cohort based upon the rationale that Melphalan cytotoxicity is enhanced 3-fold in conditions of hypoxia-induced acidosis [22] and a previous study reporting grade 3 post-perfusion neutropenia in 18% of patients treated with Melphalan-based poly-chemotherapy [4]. In our study, pelvic perfusion was immediately followed by hemofiltration to protect against toxicity, rather than the use of a pneumatic anti-shock garment [16], which modifies hemodynamic and respiratory parameters but does not prevent leakage.

Long-term MST for Melphalan mono-therapy was 37 months for IIIB patients and 19 months for IIIC patients, who previously progressed following standard therapy and surgery. MST was decreased to eight months in stage IV patients and significantly lower survival characterized the remaining 48.8% of patients who had interrupted perfusion due to disease progression, compared to patients who interrupted treatments by refusing consent or due to general worsening of conditions. One patient developed a skin reaction and mild dyspnea during the 10th Melphalan perfusion, which were resolved by corticosteroid and antihistamine treatment. Allergy to Melphalan is not common but has been reported in approximately 2% of patients [23].

MST in the wild-type *BRAF* cohort was 20 months with a median PFS of 17.7 months, and the MST of the 11 patients not eligible for target therapy or new immunotherapy was 21 months. No other therapy has demonstrated significant clinical efficacy in this wild-type *BRAF* subgroup and the only alternative therapy suggested is systemic chemotherapy. A survival benefit of >10% is required for a new therapeutic regimen or modality to be recommended. Very recently, overall survival of patients with metastatic melanoma treated with new target and immunotherapies in a real-life setting has been compared to an overall survival of 7.4 months in a 95-patients-cohort treated with systemic chemotherapy [24]. In this retrospective study, a survival benefit of >10% was recorded for our procedure in wild type *BRAF* melanoma patients, suggesting a potentially important survival benefit. However, greater numbers will be required to confirm this.

It would be interesting to determine whether Melphalan pelvic perfusion under conditions of hypoxia may generate an immune response that could be augmented by systemic immunotherapy with anti-programmed cell death-ligand protein 1 (PD-L1) antibodies [25]. In this regard, two trials are currently underway to explore the efficacy of Melphalan combined with Ipilimumab, as either adjuvant (NCT01323517) or neo-adjuvant (NCT02115243) systemic immunotherapy.

A major limitation of our study is, however, the small sample size that cannot definitively establish the true benefit of this approach in patients with wild-type *BRAF* metastatic melanoma who are not eligible or non-responsive to new immunotherapeutic drugs. However, we defend our approach as necessary in order to minimize surgical procedure variability. Finally, although hypoxic pelvic perfusion is an expensive procedure, costs are similar to those incurred by isolated limb perfusion or infusion procedures for metastatic melanoma [26].

## 4. Patients and Methods

### 4.1. Patients

This project has been performed in accordance with the Declaration of Helsinki and has been approved by the ethics committee of University of L′Aquila, L′Aquila, Italy. Written informed consent was obtained from each patient.

In this retrospective study, in order to respect performance homogeneity, a subset of patients was selected from a larger database, which included melanoma patients from different sites who underwent hypoxic perfusion. Forty-one melanoma patients with pelvic and/or inguinal loco-regional metastases, treated with a total of 175 hypoxic perfusions between September 2002 and January 2013, were included in this study. Table 3 reports patient and tumor characteristics. Patients with associated lesions below mid-thigh level, requiring a larger compartment for perfusion, were excluded from this study. Prior to treatment initiation, all patients were characterised by disease progression after previous therapies, including palliative surgery (39 patients; 95.1%), Dacarbazine-based systemic chemotherapy (19 patients; 46.3%), immunotherapy with Interferon α and/or Interleukin-2 (15 patients; 36.6%), isolated limb perfusion with tumour necrosis factor (4 patients; 9.7%), electro-chemotherapy (two patients; 4.8%), Ipilimumab (3 patients; 7.3%). Patients who had received any kind of chemotherapy, immunotherapy and/or target therapy after the last perfusion treatment were also excluded from this study. Patients with stage IV melanoma were included in this study, as loco-regional therapy was performed in these patients to avoid severe local complications. Informed consent was obtained from all patients who received complete information concerning their disease and the implications of the proposed palliative treatment, as required by the ethical standards committee on human experimentation of our institution.

### 4.2. Histopathological and Molecular Evaluation

Pathological examination revealed that all surgical specimens had an epithelioid cell pattern. Lesions were classified as “pigmented” or “non-pigmented”, based on the presence or absence of melanin-producing cells and lesions were also classified according to mitotic rate (<1 or ≥1 mitosis per mm^2^). Tumor-infiltrating lymphocytes were not detected in any of the 41 tumor specimens.

DNA was isolated from five, 10 μm formalin fixed paraffin embedded (FFPE) tissue sections from each excised lesion, using the DNA Mini Kit, as directed by the manufacturer (Qiagen, Hilden, Germany), and DNA concentration and quality determined in a Qubit fluorometer (Thermo-Fisher, Foster City, CA, USA). *BRAF* V600E mutation status was assessed using Competitive Allele Specific hydrolysis probes (TaqMan) and PCR technology (CAST) (Thermo-Fischer Scientific, Waltham, MA, USA) [29].

### 4.3. Treatment Protocol

The eligibility criteria for hypoxic pelvic perfusion have been previously reported [4]. In particular, all patients were free from renal and/or liver failure, deep venous thrombosis, severe atherosclerosis, or coagulopathy. The clinical protocol provision was for repetitive cycles of perfusion and palliative cytoreductive surgery at 6 to 7-week intervals, with purpose and timing of repetition based on previous pilot studies [4,30]. Criteria for surgical excision and other treatment details have been recently reported [25]. In the case of complete response to treatment, a prolonged treatment repetition interval of 12 weeks was performed in order to gain the clinical result of one-year progression-free survival.

### 4.4. Hypoxic Pelvic Perfusion Technique and Melphalan Regimen

All 175 perfusions were performed under general anesthesia, as previously reported [4,31]. In 123 procedures (70.3%), a percutaneous technique was adopted; in 52 treatments (29.7%) the method was surgical with 39 femoral-access, 13 iliac-access, and 49 lymphadenectomies. Details of the surgical and percutaneous techniques plus hemofiltration characteristics have been recently reported [25]. Briefly, hypoxic perfusion with hemofiltration included three phases: isolation, perfusion and hemofiltration. In the isolation phase, the blood flow to the aorta and inferior cava vein was blocked by endovascular balloon catheters and at thigh-level by pneumatic cuffs. During the perfusion phase, pelvic perfusion was performed via extracorporeal blood circulation at approximately 100 mL/min. According to previous pilot studies [4,30], 30 mg/m^2^ of Melphalan in 250 mL of isotonic sodium chloride solution was administered via the circuit over a 3-min period. The extracorporeal circuit connected to the circulation device contained a heating element and a hemofiltration module controlled by the device during perfusion and subsequent hemofiltration phases [32]. Following perfusion, balloon catheters and pneumatic cuffs were deflated to restore normal circulation and hemofiltration was then administered for 60 min (hemofiltration phase).

### 4.5. Criteria for Responses and Adverse Events

Tumor response was assessed using Response Evaluation Criteria in Solid Tumors, version 1.1 [33] at 30 days after each loco-regional chemotherapy treatment. Patient responses prior to 2009 were retrospectively re-classified. Computerized tomography (CT), Magnetic Resonance Imaging (MRI), and Position-emission Tomography (PET) were used to evaluate responses for deep masses and inspection with photo comparison employed for the monitoring of superficial lesions. Adverse events were assessed using Common Terminology Criteria for Adverse Events of the National Cancer Institute (CTCAE v4.03).

### 4.6. Statistical Analysis

Descriptive statistics estimated with 95% confidence are presented as mean ± SD Survival estimates were calculated using the Kaplan-Meier product limit estimator. No patients were lost to follow-up and no patients died of causes other than melanoma. Survival times were stratified according to clinical variables that potentially influence survival. Log-rank tests were used to assess the significant differences between the groups and hazard ratios were estimated using a proportional hazard Cox regression model. Progression free survival time (PFS) was calculated from the first day of loco-regional treatment. Statistical analyses were performed using STATA software, version 14 (Stata Corp., College Station, TX, USA).

## 5. Conclusions

In conclusion, we propose that Melphalan hypoxic perfusion is a potentially effective treatment for pelvic metastatic melanoma, but this should be confirmed in a larger multicenter prospective controlled trial.

## Figures and Tables

**Table 1:** Procedure related complications and toxicity after 175 treatments in 41 metastatic melanoma patients.

**Complications**	**Grade**	***n* (%)**
Seroma	1	4 (2.3)
Persistent leakage of fluid from the incision	2	14 (8.0)
Wound infection	1	3 (1.7)
Inguinal hematoma	1	7 (4.0)
Wound dehiscence	2	7 (4.0)
Lymphangitis	2	3 (1.7)
Scrotum edema	1	6 (3.4)
Pelvic pain	1	6 (3.4)
**Toxicity**	**Grade**	***n* (%)**
Bone marrow hypocellularity	1	25 (14.3)
	2	18 (10.3)
	3	8 (4.6)
Alopecia	1	7 (4.0)
Nausea and vomiting	1	26 (14.9)

*n* = numbers of cases.

**Table 2:** Survival according to age, gender, stage, *BRAF* V600E status, burden, mitosis, associated excision, cellular melanin pigmentation, number of treatments.

Variables (Number of Patients)	MST (Months)	Log-Rank χ^2^	*p* Value	Cox HR
Age				
<65 (*n* = 18)	17			
≥65 (*n* = 23)	20	0.80	0.371	
Gender				
Female (*n* = 28)	19.5			
Male (*n* = 13)	10	2.31	0.132	
Stage				
IIIB (*n* = 7)	37			
IIIC (*n* = 24)	19			
IV (*n* = 10)	8	21.44	0.001	4.03 [1.91–6.59]
*BRAF* status				
Wild-type (*n* = 22)	20			
V600E Mutated (*n* = 19)	13	0.36	0.551	
Burden				
Low (*n* = 23)	21			
High (*n* = 18)	16.5	7.61	0.005	2.58 [1.26–5.58]
Mitosis				
<1 (*n* = 17)	20			
≥1 (*n* = 24)	14.5	3.66	0.064	
Associate Excision				
Yes (*n* = 35)	18			
Not (*n* = 6)	17.5	2.41	0.128	
Melanin cellular pigmentation				
Yes (*n* = 15)	20			
Not (*n* = 26)	14.5	0.15	0.691	
Number of treatments				
≤2 (*n* = 7)	5			
>2 (*n* = 34)	19	1.58	0.203	

MST = median survival time; HR = hazard ratio.

**Table 3:** Characteristics of patients and tumors.

**Characteristics of Patients**		***n* (%)**
Gender	Males	13 (31.7)
	Females	28 (68.3)
Stage [27]	IIIB	7 (17.1)
	IIIC	24 (58.5)
	IV	10 (24.4)
Burden [28]	Low Burden *	23 (56.1)
	High Burden **	18 (43.9)
Patients with exclusion criteria for immune check-point therapy	Yes	8 (19.5)
	No	33 (80.5)
**Characteristics of tumors**		***n* (%)**
Anatomical site	Labia/vagina	2 (4.9)
	Anus	2 (4.9)
	Anterior trunk	2 (4.9)
	Back	3 (9.7)
	Lower extremity	31 (75.6)
Melanin presence	Yes	15 (36.6)
	No	26 (63.4)
Mitotic rate	<1 mitosis per mm^2^	17 (41.5)
	>1 mitosis per mm^2^	24 (58.5)
*BRAF* status	wild-type	22 (53.7)
	V600E mutated	19 (46.3)

*n* = numbers of patients; * <10 nodules; or no lesion >3 cm; ** ≥10 nodules; or one lesion >3 cm.

## Abbreviations

MST	median survival time
DOAJ	v-Raf murine sarcoma viral oncogene homolog B
FFPE	formalin fixed paraffin embedded
CAST	competitive allele specific technology
CT	Computerized tomography
MRI	Magnetic Resonance Imaging
PET	Position-emission Tomography
CTCAE	common Terminology Criteria for Adverse Events
SD	standard deviation
PFS	progression free survival
MAP	mitogen-activated protein
PD-1	programmed cell death protein 1
PD-L1	programmed cell death-ligand protein 1
